# Daycare attendance, breastfeeding, and risk of asthma in the prospective MoBa and DIPP birth cohorts

**DOI:** 10.1111/pai.70520

**Published:** 2026-10-22

**Authors:** Susanna Tall, Aino K. Rantala, Hanna‐Mari Takkinen, Tuuli E. Korhonen, Minna Kaila, Ketil Størdal, German Tapia, Heikki Hyöty, Riitta Veijola, Jorma Toppari, Mikael Knip, Lars C. Stene, Suvi M. Virtanen

**Affiliations:** ^1^ Research Program for Clinical and Molecular Metabolism, Faculty of Medicine University of Helsinki Helsinki Finland; ^2^ Department of Public Health Finnish Institute for Health and Welfare Helsinki Finland; ^3^ Department of Chronic Diseases Norwegian Institute of Public Health Oslo Norway; ^4^ Center for Environmental and Respiratory Health Research (CERH), Research Unit of Population Health University of Oulu Oulu Finland; ^5^ Unit of Health Sciences, Faculty of Social Sciences Tampere University Tampere Finland; ^6^ Wellbeing Services County of Pirkanmaa Tampere University Hospital Tampere Finland; ^7^ Public Health Medicine University of Helsinki Helsinki Finland; ^8^ Department of Paediatrics Tampere University Hospital Tampere Finland; ^9^ Department of Paediatric Research, Institute of Clinical Medicine, Faculty of Medicine University of Oslo Oslo Norway; ^10^ Department of Paediatric and Adolescent Medicine Oslo University Hospital Oslo Norway; ^11^ Department of Virology, Faculty of Medicine and Health Technology Tampere University Tampere Finland; ^12^ Fimlab Laboratories Tampere Finland; ^13^ Department of Pediatrics, Research Unit of Clinical Medicine, Medical Research Center University of Oulu Oulu Finland; ^14^ Department of Children and Adolescents, Medical Research Center Oulu University Hospital Oulu Finland; ^15^ Research Centre for Integrative Physiology and Pharmacology, Institute of Biomedicine University of Turku Turku Finland; ^16^ Centre for Population Health Research University of Turku, and Turku University Hospital Turku Finland; ^17^ InFLAMES Research Flagship Center University of Turku Turku Finland; ^18^ Department of Pediatrics Turku University Hospital Turku Finland; ^19^ Pediatric Research Center, New Children's Hospital Helsinki University Hospital Helsinki Finland; ^20^ Centre for Child Adolescent and Maternal Health Research (TamCAM), Tampere University Tampere Finland

**Keywords:** asthma, breastfeeding, cohort study, daycare, DIPP, MBRN, MoBa

## Abstract

**Background:**

Daycare attendance and breastfeeding have been associated with asthma risk. However, it remains unclear whether the association between breastfeeding and asthma risk depends on daycare attendance. We investigated whether daycare attendance acts as a modifier in the relationship between breastfeeding and asthma risk.

**Methods:**

We included 72,558 children from the MoBa and 3015 children from the DIPP studies. Current asthma at age 7 years in MoBa and time to asthma by the age 5 years in DIPP were analyzed.

**Results:**

In both cohorts, approximately 4% of participants were diagnosed with asthma. In MoBa, breastfeeding was associated with lower risk of overall asthma (adjusted risk ratio, aRR 0.99 [95% CI, 0.98–1.00]) and nonatopic asthma (aRR 0.97 [95% CI, 0.96–0.99]) in children who had attended daycare by the age of 18 months, and it was associated with higher overall asthma (aRR 1.01 [95% CI, 1.00–1.02]) and atopic asthma risk (aRR 1.03 [95% CI, 1.01–1.06]) in children who had not attended daycare by that age (all interaction *p* values ≤.05). In DIPP, the duration of breastfeeding was inversely associated with risk of atopic asthma only in an exploratory analysis in those who had started daycare by the age of 12 months (adjusted hazard ratio, aHR 0.75 [95% CI, 0.59–0.95]) but not in children who had started daycare at the age of 13–24 months (adjusted hazard ratio, aHR 1.03 [95% CI, 0.96–1.10]) or had not started daycare by the age of 24 months (adjusted hazard ratio, aHR 1.00 [95% CI, 0.94–1.06]) (interaction *p* value .04).

**Conclusions:**

While the primary analysis in the MoBa study suggested that age at daycare entry might mildly modify the association of breastfeeding with asthma risk, in the DIPP study an interaction only appeared in an exploratory analysis with limited atopic asthma cases. Consequently, these findings represent inconsistent evidence.

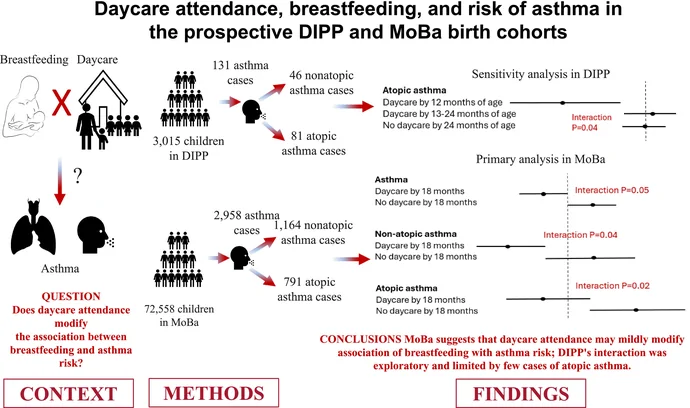

## Graphical abstract

The findings obtained in this study report inconsistent signals rather than systematic evidence that early daycare attendance modifies the effect of breastfeeding to protect against asthma.

**Key message**

This study reports inconsistent signals rather than systematic evidence that early daycare attendance modifies the association between breastfeeding and asthma risk. Primary analyses in the large MoBa cohort suggest a mild modification by age at daycare entry; however, this interaction was not robustly replicated in the DIPP cohort, emerging only in exploratory sub‐analyses with limited atopic asthma cases.

## 1 INTRODUCTION

According to the World Health Organization and the Global Burden of Disease study, asthma affects approximately 260 million people worldwide.^1^ Annual national direct costs for treating asthma, allergic rhinitis, and eczema of subjects up to 44 years of age are approximately EUR 83 million in Norway alone.^2^ Despite the substantial economic burden of modern therapeutics, optimal asthma control remains elusive for many patients. This persistent symptom burden inflicts continuous distress, particularly on affected young children.

Asthma diagnosis is based on recurring symptoms, including easily triggered bronchospasms and reversible airflow obstruction. The disease has traditionally been further classified based on underlying mechanisms of nonatopic asthma and atopic asthma.^3^ Nonatopic asthma originates from triggers that do not include an immune response to an allergen, such as viral infections. Atopic asthma is caused by allergen‐induced unfavorable immune responses initiating various symptoms in different organs.

While the underlying pathophysiology and some genetic risk factors of asthma have been established, our understanding of the environmental triggers remains incomplete.^4^, ^5^ The original hygiene hypothesis stated that rates of allergic diseases are increasing among children because of reduced exposure to infections.^6^ Exposure to infections during critical early‐life windows is thought to calibrate regulatory immune networks, ensuring proper immunological development. On the other hand, several studies have reported that lower respiratory tract infections are associated with increased risk of asthma in children.^7^ More recent views of the hygiene hypothesis stress that fewer social contacts reduce the total microbial exposure, not just symptomatic infections, preventing the immune system from developing tolerance, which in turn leads to allergies and autoimmune manifestations.^8^

Daycare attendance increases peer‐to‐peer contact, resulting in a significantly more varied microbial exposure than is typically observed in home environments, e.g.^9^, ^10^, ^11^ If the modern view of the hygiene hypothesis holds true, a reduction in asthma incidence should correlate with daycare attendance. However, the literature in relation to this topic is conflicting. A meta‐analysis of 18 studies found that early daycare attendance is associated with an increased risk of asthma in 3‐ to 5‐year‐olds but not later.^12^ On the other hand, a large Norwegian prospective cohort study, which was not included in the previously mentioned meta‐analysis, reported a protective association of daycare with risk of overall asthma.^13^ Furthermore, some studies have reported a decreased probability of late‐onset wheezing^14^ and atopy^15^ with daycare attendance.

Breastfeeding also contributes to the development of the immune system and may affect risk of asthma.^16^, ^17^ By interacting with microbial exposure in a child, breastfeeding may modify the incidence of allergic diseases together with daycare attendance. However, the interaction of social mixing and breastfeeding on risk of allergic diseases has not been established in scientific research. Some research suggests that breastfeeding is associated with lower risk of early wheezing and the non‐atopic asthma phenotype, though the association with asthma after early childhood is unclear.^17^, ^18^, ^19^

To our knowledge we are the first to address the interaction between daycare attendance and breastfeeding on risk of overall asthma, non‐atopic asthma, and atopic asthma. We also compared the incidence of different asthma subtypes in children attending daycare and those not attending daycare, and the association between daycare attendance and breastfeeding on the risk of asthma subtypes. Based on previously published studies we expected that daycare attendance would be associated with reduced asthma risk. Furthermore, breastfeeding was expected to decrease the risk of asthma in children. Foremost, we hypothesized that the association between breastfeeding and asthma risk would be boosted by early daycare attendance and that this would be seen as a significant interaction between the two.

## 2 METHODS

### 2.1 Study populations

This study used data from two birth cohort studies: the Norwegian Mother, Father and Child Cohort Study (MoBa) and the Finnish Type 1 Diabetes Prediction and Prevention (DIPP) study. Written informed consent was obtained from parents or legal guardians for children, with assent typically sought from the children themselves depending on age and maturity.

### 2.2 MoBa

MoBa is a prospective population‐based study that includes approximately 114,500 children and their parents conducted by the Norwegian Institute of Public Health. Women were recruited throughout Norway at approximately 18 weeks of gestation between 1999 and 2008.^20^ The participation rate of this total population was 41%. We included all children in MoBa that were still alive and living in Norway, with information from the Medical Birth Registry of Norway (MBRN). MBRN is a national health registry containing information about all births in Norway. Children with prospective daycare exposure data from birth to at least 18 months of age, data on breastfeeding duration, and asthma diagnosis at the age of 7 years were included. Children who did not participate in the 18‐month follow‐up were excluded from the study, leaving a total of 72,558 children in the overall asthma study population (Figure S1).

The definition of asthma was based on dispensed asthma medications from the Norwegian Prescription Database,^21^ as in previous studies of asthma in MoBa e.g.^22^ Asthma at age 7 years was defined as having at least two dispensed prescriptions for asthma medications: one in the 12 months preceding the seventh birthday in addition to a second dispensed prescription within 12 months of the first. Asthma medications included inhaled beta_2_‐agonists (ATC code R03AC), inhaled glucocorticoids (R03BA), combinations of inhaled beta_2_‐agonists and glucocorticoids (R03AK), and leukotriene receptor antagonists (R03DC). A child's current atopy at 7 years was defined as atopic dermatitis, animal allergy, or allergic rhinitis reported by the parent to have been verified by a doctor in questionnaires administered at age 7 years. A child with asthma diagnosed with atopy was categorized as having atopic asthma. If the parents reported that the child did not have current atopic dermatitis, animal allergy, or allergic rhinitis verified by a doctor, the asthma diagnosis was categorized as non‐atopic asthma. If the family did not return in the 7‐year questionnaire, the children were excluded from the study of atopic and non‐atopic asthma subtypes.

We obtained information on daycare attendance when the child was 18 months old from questionnaires.^23^ The mother was asked where and how her child was cared for during the day, in age intervals 0–6 months, 7–9 months, 10–12 months, 13–15 months, and 16–18 months. The listed response categories included “at home with a parent” and “at home with a childminder,” which we categorized as home care, and “at a childminder's / in a family day nursery” and “in a daycare center,” which we categorized as family daycare and daycare center, respectively. Any period in family daycare or a daycare center before 18 months of age (occasional or regular attendance) was regarded as attendance at daycare. During exclusive breastfeeding, an infant could receive water, vitamins, mineral drops, or medicines in addition to breast milk. The duration of overall breastfeeding was defined as any breast milk provided. MoBa is regulated by the Norwegian Health Registry Act. The current study was approved by the Regional committees for medical and health research ethics (175814).

### 2.3 DIPP

The research was accomplished using the Finnish DIPP study data, which includes children with human leukocyte antigen (HLA) genotypes associated with an increased risk of type 1 diabetes. In DIPP, more than 250,000 infants have been screened for HLA genotypes, of which a total of approximately 25,000 children have started clinical follow‐up. At the age of 5 years, all children participating in DIPP who were born between September 2, 1996 and September 5, 2004 in Oulu and Tampere University Hospital areas were invited to take part in an allergy study. Of the 4075 children invited, 3781 children participated. The parents of 3143 children completed a form regarding allergic diseases and asthma in the participant's family. Altogether, the analysis included 3015 children with prospective daycare exposure data from birth to at least 24 months of age, alongside complete data on both total breastfeeding duration and asthma status between ages 2 and 5 (Figure S2).

A questionnaire modified from the International Study of Asthma and Allergies in Childhood's (ISAAC) questionnaire on the child's history of allergic symptoms and asthma was completed by the families.^24^ Asthma was defined as doctor‐diagnosed asthma plus either any wheezing symptom or use of asthma medication during the preceding 12 months. Age of the child at asthma diagnosis was determined by the following question: “At what age was asthma diagnosed?” Atopic eczema was defined as atopic eczema diagnosed by a doctor. Specific IgE concentrations were analyzed from blood samples by using the ImmunoCAP fluoroenzyme immunoassay (Phadia Diagnostics, Uppsala, Sweden) for the following food and inhalant allergens: egg, cow's milk, fish, wheat, house dust mite, cat, timothy grass, and birch. Atopy was defined as sensitization (≥0.35 kU/L) to any of the tested allergens. If a child with asthma was diagnosed with atopy, then the asthma diagnosis was categorized as atopic asthma. Nonatopic asthma was defined as asthma in children who were IgE‐negative. Participants were asked whether daycare attendance started by the age of 12, 24, or 36 months (yes/no) in the modified ISAAC questionnaire. During exclusive breastfeeding, in addition to breast milk an infant could receive water, vitamins, mineral drops, or medicines. The duration of overall breastfeeding was defined as any breast milk provided. Food records collecting diet and daycare data were completed at 3, 6, 9, 12, 15, and 24 months and then annually thereafter.

### 2.4 Statistical analyses

In both the MoBa and DIPP data, we tested the statistical significance of interaction between a dichotomous daycare attendance variable and a continuous total duration of breastfeeding variable in separate regression models with asthma, non‐atopic asthma, or atopic asthma as the outcomes.

#### 2.4.1 MoBa

The Stata software was used to analyze the MoBa data. As this cohort utilized a registry‐based design where exact event dates were unavailable but the cross‐sectional point prevalence at age 7 was available, a relative risk (RR) approach was the mathematically appropriate choice to evaluate the relative risk at that fixed time point. We assessed the interaction between a dichotomous daycare attendance variable (no daycare vs. daycare attendance by 18 months of age) and the total duration of breastfeeding, which was modeled as a continuous variable representing 1‐month increments. Confounders are listed in Appendix S1. The risk ratios (RRs) of asthma and 95% CIs were estimated using log‐linear binomial regression models with robust variance estimation. Altogether, 2941 of the 72,558 children in the study population (4.1%) had missing information on at least one of the confounders. No data imputation was carried out in MoBa as data missingness did not exceed 5%.^25^

#### 2.4.2 DIPP

The SAS Enterprise Guide version 8.3 (SAS Institute Inc.) statistical software package was used for the statistical analysis of DIPP data. As DIPP cohort had longitudinal, precise tracking of when asthma occurred (e.g., specific dates of diagnosis) alongside varying lengths of individual follow‐up a Cox proportional hazards model was used to leverage this granular time data and account for censoring over time. The interaction was established between a dichotomous daycare attendance variable (daycare vs. no daycare by 24 months of age) and the total duration of breastfeeding, modeled as a continuous variable per 1‐month increment. Confounders are listed in Appendix S1. Altogether 700 (23.2%) of the 3015 children in the study population had missing information on at least one of the confounders. We imputed the missing values using chained equations (MICE) with 20 imputations^26^ and present the results from the multiple imputed dataset and unadjusted dataset. Statistical estimates and covariance matrices across all 20 imputed datasets were pooled using Rubin's rules.

### 2.5 Sensitivity analyses

To test the robustness of the findings, some sensitivity analyses were carried out with both the DIPP and MoBa datasets. Firstly, interaction between daycare attendance by 36 months vs. no daycare attendance by 36 months and the total duration of breastfeeding was analyzed. Furthermore, interaction between exclusive breastfeeding and daycare attendance vs. no daycare attendance was analyzed (daycare attendance by 24 months vs. no daycare attendance by 24 months in DIPP and daycare attendance by 18 months vs. no daycare attendance by 18 months in MoBa). Additionally, a sensitivity analysis additionally adjusting for the continuous household income variable as a marker of socioeconomic status (SES) was conducted. Because comparable income data were not available in the DIPP cohort, we restricted this sensitivity analysis to the MoBa cohort.

Furthermore, in DIPP, interaction between daycare attendance by the age of 12 months, daycare attendance at the age of 13–24 months, and no daycare by the age of 24 months and the total duration of breastfeeding was analyzed. Due to the multi‐degree‐of‐freedom nature of the interaction term, a global, pooled multivariate Wald Chi‐square test (2 degrees of freedom) was utilized to evaluate the overall significance of the interaction. Pairwise stratum‐specific hazard ratios (HR) and 95% confidence intervals (CI) for total breastfeeding within each of the three distinct categories of daycare attendance were planned for extraction only upon a significant global interaction test (*p* < .05).

### 2.6 Absolute risks

To enhance clinical interpretability, multivariable‐adjusted relative risk estimates for significant interactions were translated into absolute risk trajectories over a 3‐month continuous breastfeeding exposure contrast. For the MoBa cohort, these absolute profiles and their 95% confidence intervals (CIs) were generated using adjusted predictive margins to establish group baselines at the cohort mean, combined with average marginal effects to model the continuous trajectory shifts. Because the DIPP datasets were processed using MICE (20 imputed datasets), calculating accurate pooled absolute risk trajectories presented profound technical limitations. To maintain the highest level of statistical rigor, we have restricted the DIPP reporting solely to the highly robust pooled multivariate HRs per 1‐month breastfeeding increment, which are structurally suited for MICE integration.

## 3 RESULTS

### 3.1 MoBa

Of the 72,558 children included in the MoBa data, 2958 (4.1%) had asthma at 7 years of age. Information on atopy at the age of 7 years was obtained for 47,032 children. Of these children, 1164 (2.5%) were diagnosed with nonatopic asthma and 791 (1.7%) with atopic asthma. Approximately 71% of the children attended daycare by the age of 18 months. The included MoBa children were breastfed for an average of 9.9 months (SD 4.5). Table 1 presents the baseline characteristics of the cohort participants stratified by daycare attendance, alongside the corresponding mean duration of breastfeeding.

**TABLE 1:** Distribution of maternal and child characteristics according to daycare attendance and breastfeeding duration in the MoBa and DIPP study cohorts.

	MoBa (*N* = 72,558)			DIPP (*N* = 3015)^a^		
	Daycare attendance by 18 months of age (%)		Breastfeeding duration, months mean (SD)	Daycare attendance by 24 months of age (%)		Breastfeeding duration, months mean (SD)
	No	Yes		No	Yes	
Maternal body mass index						
<18.5 underweight	726 (3)	1365 (3)	9.8 (4.6)	55 (4)	41 (3)	8.3 (5.3)
18.5–24.9 normal weight	12,894 (61)	33,756 (66)	10.3 (4.3)	906 (58)	891 (61)	8.0 (4.9)
25–29.9 overweight	4620 (22)	10,679 (21)	9.5 (4.7)	442 (28)	399 (27)	7.8 (5.5)
>30 obesity	2169 (10)	4196 (8)	8.4 (5.1)	157 (10)	124 (9)	6.9 (5.3)
Missing	736 (3)	1417 (3)	9.7 (4.6)			
Maternal age at delivery						
<25 years	2613 (12)	4173 (8)	7.9 (4.5)	248 (16)	197 (14)	6.3 (4.5)
25–29 years	6686 (32)	17,335 (34)	9.6 (4.3)	487 (31)	565 (39)	7.4 (4.7)
30–34 years	7698 (36)	21,126 (41)	10.3 (4.4)	500 (32)	436 (20)	8.3 (5.0)
35 years or more	4148 (20)	8779 (17)	10.8 (4.7)	325 (21)	257 (18)	9.2 (6.3)
Maternal education^b^						
1 (least educated)	10,666 (50)	36,539 (71)	10.6 (4.2)	109 (7)	44 (3)	5.5 (4.5)
2	10,183 (48)	14,375 (28)	8.5 (4.7)	476 (30)	313 (22)	6.9 (5.3)
3	–	–	–	730 (47)	672 (46)	7.9 (4.8)
4	–	–	–	245 (16)	424 (29)	9.5 (5.3)
Missing	296 (1)	499 (1)	9.6 (4.6)			
Maternal smoking during pregnancy						
Non‐smoking	14,591 (69)	37,527 (73)	10.4 (4.4)	1441 (92)	1341 (92)	8.1 (5.2)
Previous smoking	1156 (5)	3051 (6)	9.6 (4.3)	–	–	–
Stopped by 18 gestational weeks	2789 (13)	6824 (13)	9.0 (4.5)	21 (1)	13 (1)	5.8 (4.7)
Continued after 18 gestational weeks	2381 (11)	3581 (7)	7.6 (4.8)	98 (6)	101 (7)	5.4 (4.6)
Missing	228 (1)	430 (1)	10.3 (4.5)			
Maternal asthma						
No	19,391 (92)	47,440 (92)	9.9 (4.5)	1415 (47)	1329 (44)	7.9 (5.1)
Yes	1551 (7)	3655 (7)	9.6 (4.7)	145 (5)	126 (4)	8.0 (5.6)
Missing	203 (1)	318 (1)	9.8 (4.6)			
Delivered by cesarean section						
No	17,806 (84)	44,242 (86)	10.1 (4.4)	1362 (87)	1253 (86)	8.0 (5.1)
Yes	3339 (16)	7171 (14)	9.0 (4.9)	198 (13)	202 (14)	7.1 (5.5)
Number of previous deliveries						
0	8150 (39)	25,681 (50)	9.6 (4.5)	588 (38)	760 (52)	7.4 (5.0)
1	6886 (33)	18,228 (35)	9.9 (4.4)	466 (30)	476 (33)	7.7 (5.0)
2 or more	6109 (29)	7504 (15)	10.7 (4.5)	506 (32)	219 (15)	9.1 (5.6)
Born preterm (<37 weeks)						
No	19,402 (92)	48,634 (95)	10.0 (4.5)	1493 (96)	1406 (97)	8.0 (5.2)
Yes	1653 (8)	2585 (5)	8.6 (4.9)	67 (4)	49 (3)	5.8 (5.2)
Missing	90 (0.4)	194 (0.4)	9.7 (4.4)			
Atopic dermatitis at 6 months						
Yes	2560 (12)	6083 (12)	9.8 (4.5)	168 (11)	202 (14)	7.6 (5.0)
No	18,585 (88)	45,330 (88)	9.9 (4.5)	1392 (89)	1253 (86)	7.9 (5.2)
Sex						
Boy	10,856 (51)	26,228 (51)	9.8 (4.5)	816 (52)	762 (52)	7.8 (5.2)
Girl	10,289 (49)	25,185 (49)	10.0 (4.5)	744 (48)	693 (48)	8.0 (5.2)

Abbreviations: DIPP, Type 1 Diabetes Prediction and Prevention study (Finland); MoBa, Norwegian Mother, Father and Child Cohort Study.

^a^ Rounded average cell counts, percentages, mean values, and standard deviations (SDs) are shown based on imputed data.

^b^ Maternal education categories: in MoBa (1) high school or less, (2) some college studies; in DIPP (1) no vocational education or university degree, (2) vocational school or course, (3) secondary vocational education, (4) university studies or degree.

A significant but weak interaction between daycare attendance and breastfeeding was observed for all asthma outcomes but only when the analyses were adjusted for confounders (Figure 1, Table S1); while the relative risk calculations model each 1‐month increment of breastfeeding duration, the corresponding absolute risk projections utilize a 3‐month scale. Breastfeeding provided some minimal protection against overall asthma and nonatopic asthma when a child attended daycare by the age of 18 months. It was also associated with a minimally increased overall asthma and atopic asthma risk when a child did not attend daycare (Figure 1). The adjusted baseline absolute risks closely mirror the observed sample prevalence across all exposure strata (ranging from 1.61% for atopic asthma to 4.61% for overall asthma, Table S2). Consequently, a 3‐month step‐increase in breastfeeding duration (representing three compounded 1‐month relative increments) yields nominal absolute risk trajectories, shifting the adjusted risk by a maximum of 0.19 percentage points across all categories. For example, a 3‐month continuous step‐increase shifts overall asthma risk by just 0.10 percentage points in either direction—moving from 4.61% to 4.71% in the home‐care group, and down from 3.85% to 3.75% in the early daycare group.

**FIGURE 1:**
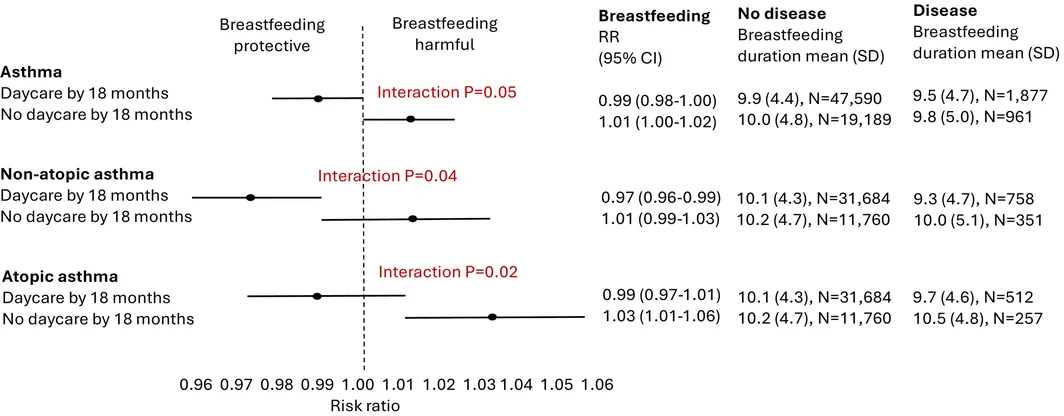
Interaction between daycare attendance by 18 months of age and total duration of breastfeeding on the risk of developing asthma, nonatopic asthma, and atopic asthma in the Norwegian Mother, Father and Child Cohort Study (MoBa). Log‐linear binomial regression models were adjusted for the main effects of breastfeeding duration and daycare attendance, their interaction term, as well as maternal pre‐pregnancy BMI, age, education, smoking, asthma, c‐section, parity, child's premature birth, eczema by the first 6 months, and sex. CI, confidence interval; SD, standard deviation.

In our sensitivity analyses, duration of exclusive breastfeeding did not interact with daycare attendance by 18 months (Table S3) nor was the total duration of breastfeeding interactively associated with daycare attendance at the age of 36 months (Table S4) in the MoBa study cohort. The inclusion of household income as an additional confounder in a sensitivity analysis only marginally altered the effect estimates but did not change the statistical significance of the findings (Table S5).

Daycare attendance by the age of 18 months was associated with reduced risk of overall asthma and both asthma subtypes in MoBa both with and without adjustment for confounders (all *p* values <.05, Table S6). Breastfeeding was marginally associated with lower risk of non‐atopic asthma (unadjusted RR 0.97 [95% CI, 0.96–0.98], *p* < .0001, fully adjusted RR 0.98 [95% CI, 0.97–1.00], *p* = .02) but not with overall asthma or atopic asthma (Table S6).

### 3.2 DIPP

Of the 3015 children included in the DIPP data, 131 (4.3%) developed asthma by the age of 5 years. Of these, 81 (2.7%) were further diagnosed with atopic asthma and 46 (1.5%) with nonatopic asthma, while four asthma cases could not be clinically categorized due to missing sub‐phenotypic data. Approximately 48% of the included children attended daycare by the time of their second birthday. The included DIPP children were breastfed on average for 7.9 months (SD 5.2). Table 1 presents the baseline characteristics of the cohort participants stratified by daycare attendance, alongside the corresponding mean duration of breastfeeding.

While binary daycare attendance by the age of 24 months did not modify the association between breastfeeding and any of the three asthma outcomes (Table 2, Table S7), a more granular timing sensitivity analysis revealed a significant interaction for atopic asthma (Figure 2, Table S8), with all effect estimates and risk parameters modeled per single 1‐month increment of breastfeeding duration. Specifically, the inverse association between breastfeeding duration and atopic asthma risk was restricted to children who entered daycare by 12 months of age. In contrast, breastfeeding was not associated with risk of atopic asthma among children who started daycare between 13 and 24 months of age, or those who did not attend daycare by 24 months (Figure 2, Table S8). The association between breastfeeding and atopic asthma risk in the group that attended daycare by the age of 12 months differed significantly from the group that did not attend daycare by the age of 24 months (interaction *p* = .02), whereas the later‐entry group (13–24 months) showed no such difference compared with this reference group (interaction *p* = .56). Finally, daycare attendance by 24 months did not modify the association between exclusive breastfeeding and asthma risk (Table S3), nor did total breastfeeding duration interact with daycare attendance by 36 months (Table S4).

**TABLE 2:** Interaction between daycare attendance, duration of breastfeeding, and risk of developing asthma subtypes: Adjusted analyses in the DIPP study cohort. Daycare attendance was defined as any outside home care by the age of 24 months.

	No disease	Disease	Adjusted hazard ratio (95% CI)^a^ for breastfeeding	*p* value for daycare and breastfeeding interaction^a^
	Breastfeeding duration, months mean (SD)	Breastfeeding duration, months mean (SD)		
Asthma				
Daycare	7.4 (4.8), *N* = 1393	6.5 (3.8), *N* = 62	0.97 (0.91–1.03)	.85
No daycare	8.4 (5.5), *N* = 1491	7.4 (5.4), *N* = 69	0.97 (0.93–1.02)	
Nonatopic asthma				
Daycare	7.4 (4.8), *N* = 1393	5.6 (3.9), *N* = 18	0.91 (0.80–1.03)	.53
No daycare	8.4 (5.5), *N* = 1491	6.4 (4.2), *N* = 28	0.95 (0.88–1.03)	
Atopic asthma				
Daycare	7.4 (4.8), *N* = 1393	7.0 (3.7), *N* = 44	0.99 (0.92–1.06)	.82
No daycare	8.4 (5.5), *N* = 1491	8.3 (6.4), *N* = 37	1.00 (0.94–1.06)	

Abbreviations: CI, confidence interval; DIPP, Type 1 Diabetes Prediction and Prevention study (Finland); SD, standard deviation.

^a^ Adjusted for total duration of breastfeeding, daycare attendance, and their interaction, as well as maternal pre‐pregnancy BMI, age, education, smoking, asthma, c‐section, parity, child's premature birth, eczema by the first 6 months, and sex.

**FIGURE 2:**
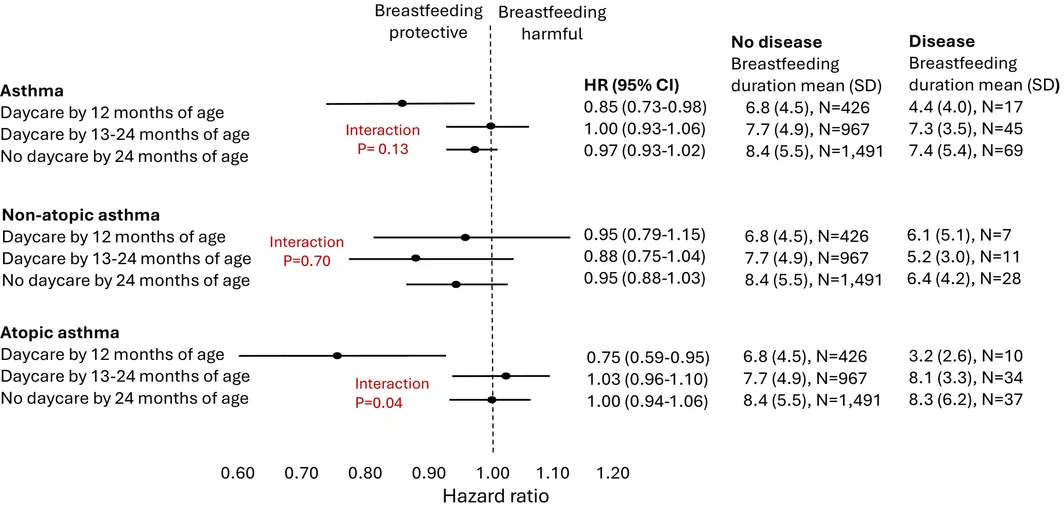
Association between total duration of breastfeeding and asthma stratified by three levels of daycare attendance in the Finnish Type 1 Diabetes Prediction and Prevention (DIPP) study cohort. Cox regression models were adjusted for the main effects of breastfeeding duration and daycare attendance, their interaction term, as well as maternal pre‐pregnancy BMI, age, education, smoking, asthma, c‐section, parity, child's premature birth, eczema by the first 6 months, and sex. CI, confidence interval; SD, standard deviation.

Daycare attendance by the age of 24 months was not associated with any of the asthma outcomes in DIPP (Table S9). In the unadjusted analyses, each 1‐month increment in total breastfeeding duration was marginally associated with lower risk of overall asthma (HR, 0.96; 95% CI, 0.93–1.00; *p* = .05) and nonatopic asthma (HR, 0.92; 95% CI, 0.86–0.99; *p* = .02) but not atopic asthma (HR, 0.99; 95% CI, 0.95–1.03; *p* = .56). After adjustment for confounders, the total duration of breastfeeding showed a marginal, nonsignificant inverse association per 1‐month increment with risk of overall asthma (HR, 0.97; 95% CI, 0.93–1.01; *p* = .12) and nonatopic asthma (HR, 0.94; 95% CI, 0.87–1.00; *p* = .06).

## 4 DISCUSSION

Contrary to our primary hypothesis, the findings obtained in this study report inconsistent signals rather than systematic evidence that early daycare attendance modifies the association of breastfeeding with asthma risk. While the findings obtained in the MoBa study suggest that the association between breastfeeding and asthma risk may be modified by the age at daycare entry, the primary binary daycare analyses in DIPP were negative. In the DIPP, the reported interaction emerged only in a three‐category exploratory analysis based on few cases of atopic asthma. Furthermore, our absolute risk calculations suggest that the clinical relevance of the observed interactions is limited in MoBa.

The modern hygiene hypothesis argues that immunologic protection in the natural history of allergic diseases, including asthma, stems from early contact with a variety of microbes,^8^ but this theory does not consider the potential role of breastfeeding. Indeed, recent studies suggest that breastfeeding may directly or indirectly interact with microbes in an infant.^27^ The composition of breast milk may change when an infant is sick and become richer in infection‐fighting compounds such as lymphocytes, which in turn may help the infant to recover. Furthermore, breastfeeding may provide long‐term protection against various infections by transferring antibodies, immune cells, and growth factors to the child, which can support the development of the immune system in infancy.^28^ Breastfeeding may provide immune support to fight off infections, while the microbe‐rich environment in daycare environments prevents the development of immune reactions that lead to asthma. Nevertheless, our findings only partially and inconsistently align with this conclusion.

While our findings tentatively suggest that early daycare entry may influence the relationship between breastfeeding and microbial exposure, further studies and microbial data are needed to confirm this link. While breast milk has immune‐modulating properties, its ability to prevent asthma in offspring has been shown to depend on the gut microbiota in recent mouse experiments.^29^, ^30^ In mice raised in a sterile environment, breast milk fails to stimulate immune tolerance, leading to persistent Th2‐dependent allergic airway responses in the offspring. However, a protective association was observed in a non‐sterile environment where gut microbes were able to interact with the compounds present in breast milk.^30^ Thus, the protective benefits of breast milk rely on microbial presence, at least in mice. Because of uncertainty regarding the interpretation of results from mouse models, further human studies remain crucial to make any generalizations.

Lack of microbial exposure together with breastfeeding could hypothetically result in modulations in immune system development that may in turn explain why breastfeeding was associated with mildly increased asthma and nonatopic asthma risks in children who had not attended daycare by the age of 18 months in MoBa. However, this is speculative at best, as in DIPP no such association was observed. Some studies have reported an increased risk of asthma with breastfeeding in children of mothers that are affected by asthma.^31^ However, some other studies have not found such an association.^32^ Furthermore, certain factors can link breastfeeding to a higher risk of offspring allergic diseases. These include high pollutant exposure^33^ and less diverse breast milk microbiota.^34^ In line with our observations, scientific literature thereby shows that breastfeeding does not uniformly protect infants from allergic diseases but may in some specific circumstances increase the risk of such conditions.

Although the effect of the interaction between daycare attendance and breastfeeding on risk of asthma has not been investigated before, protective interaction between these variables was found in relation to type 1 diabetes in a small prospective cohort study (Diabetes Autoimmunity Study in the Young, DAISY) in Colorado, USA.^35^ In that study, daycare attendance increased the risk of type 1 diabetes in non‐breastfed children and provided increasing protection in the case of longer duration of breastfeeding. While type 1 diabetes and asthma are different diseases, they do have a lot in common. Type 1 diabetes and asthma both fall under the theoretical framework of the hygiene hypothesis and have shown similar increases in incidence and prevalence over time, suggesting common environmental triggers.^36^ They have a complex relationship where one condition can change the risk of the other and the conditions are both immune‐mediated.^37^ Even so, meta‐analyses and Mendelian Randomization (MR) studies demonstrate that genetic variants driving type 1 diabetes, predominantly located in the human leukocyte antigen (HLA) region, do not significantly increase the risk of asthma, indicating that the genetic backgrounds of these conditions largely diverge.^38^

The observed interaction between daycare attendance and breastfeeding may explain the variable findings reported across prior studies investigating the association between breastfeeding and asthma risk. If breastfeeding and daycare attendance are not evaluated simultaneously, potential associations may remain masked. A large meta‐analysis of 117 studies reported a protective association between breastfeeding and asthma risk. However, a high level of heterogeneity in the effect sizes between the included studies was observed.^17^ That review concluded that wheezing—which often leads to non‐atopic asthma in infants—is predominantly triggered by viral respiratory infections, against which breastfeeding provides immunological protection. As a child ages, confounding environmental and host factors increasingly influence respiratory and immunologic morbidity, obscuring the isolated impact of breastfeeding. However, because the development of later‐onset asthma is heavily mediated by early‐life infections, the early protective effects of breastfeeding against infant infections likely explain its sustained benefit into childhood.

Our study has several strengths. Firstly, to our knowledge we are the first to address whether daycare attendance may modify the association of breastfeeding with risk of asthma. Secondly, the data were collected prospectively and independently in two different countries, increasing the generalizability and accuracy of the data. Thirdly, MoBa data included an impressive number of children (72,558). The small number of children from DIPP may limit the conclusions that can be drawn from the data. We adjusted our analyses for several well‐known confounders. However, residual confounding due to some unknown or unmeasured variables can never be excluded in an observational study. As specific demographic or clinical data for the 1060 invited but non‐included children were unavailable for direct statistical comparison in DIPP, we acknowledge the lack of characterization for these non‐participants as a limitation of our study. All DIPP participants carried increased HLA‐susceptibility to type 1 diabetes, which limits the generalizability of the DIPP data. Furthermore, it should be acknowledged that our multiple interaction and sensitivity analyses were exploratory and were not adjusted for multiplicity. Consequently, these secondary findings should be interpreted with caution and treated as hypothesis‐generating. Effect sizes observed in many of our interaction models are modest, and several tests for association modification barely reached statistical significance. Consequently, the public health impact and absolute clinical benefit remain speculative, and these findings should be interpreted strictly as exploratory signals rather than definitive evidence of an interaction.

Although the MoBa study suggests that the relationship between breastfeeding and asthma risk may mildly vary by the age of daycare entry, this interaction was not convincingly replicated in DIPP, emerging only within a three‐category exploratory framework with very few atopic asthma cases. Furthermore, our absolute risk calculations suggest that the clinical relevance of the observed interactions is limited in MoBa. Consequently, these findings represent inconsistent signals rather than robust evidence that early daycare attendance alters the association of breastfeeding with asthma risk.

## AUTHOR CONTRIBUTIONS

**Susanna Tall:** Conceptualization; investigation; writing – original draft; validation; visualization; writing – review and editing; formal analysis; data curation; funding acquisition. **Aino K. Rantala:** Writing – review and editing; formal analysis. **Hanna‐Mari Takkinen:** Methodology; validation; writing – review and editing; formal analysis; data curation. **Tuuli E. Korhonen:** Data curation; methodology. **Minna Kaila:** Data curation; methodology. **Ketil Størdal:** Methodology; data curation. **German Tapia:** Methodology; data curation; writing – review and editing. **Heikki Hyöty:** Conceptualization; investigation; methodology; project administration. **Riitta Veijola:** Methodology; conceptualization; investigation; project administration; data curation; resources. **Jorma Toppari:** Conceptualization; investigation; methodology; project administration; data curation; resources. **Mikael Knip:** Conceptualization; methodology; validation; project administration; supervision; resources. **Lars C. Stene:** Conceptualization; investigation; methodology; validation; writing – review and editing; formal analysis; project administration; data curation; supervision; resources. **Suvi M. Virtanen:** Conceptualization; investigation; methodology; validation; project administration; data curation; supervision; resources.

## FUNDING INFORMATION

This study has received funding from the European Union's Horizon 2020 research and innovation programme under grant agreement (HEDIMED) (grant 874864), the Research Council of Finland (grants 292538, 339922, and 374703), Breakthrough T1D (formerly JDRF) (grants 1‐SRA‐2016‐342‐M‐R, 1‐SRA‐2019‐732‐M‐B, 3‐SRA‐2020‐955‐S‐B, and 3‐SRA‐2023‐1433‐S‐B), the European Commission (grant BMH4‐CT98‐3314), the Novo Nordisk Foundation, the Leona M., and Harry B. Helmsley Charitable Trust (grant G‐2205‐05255), the Centre of Excellence in Molecular Systems Immunology and Physiology Research 2012–2017 (grant 250114), Special Research Funds for University Hospitals in Finland, the Diabetes Research Foundation, Finland, the Sigrid Juselius Foundation, Finland, the Foundation for Pediatric Research in Finland, the University of Helsinki Doctoral School, and the Björkqvist Foundation.

## CONFLICT OF INTEREST STATEMENT

The authors declare that they have no relevant conflicts of interest.

## Supporting information


Appendix S1.

## Data Availability

The data analyzed in the current study are not publicly available due to the general data protection legal regulations and the sensitive nature of the personal information.
