# Supplementary material for: Daycare attendance, breastfeeding, and risk of asthma in the prospective MoBa and DIPP birth cohorts
Source: Pediatr Allergy Immunol. 2026 Oct 22;37(10):e70520. doi: 10.1111/pai.70520 (PMC13691403; doi:10.1111/pai.70520)
Supplement: Supplementary file 1 [file PAI-37-e70520-s001.docx]

Online only supplementary appendix to

**Daycare attendance, breastfeeding and risk for asthma in**

**the prospective DIPP and MoBa birth cohorts**

by Susanna Tall, Aino K. Rantala, Hanna-Mari Takkinen, Tuuli E. Korhonen, Minna Kaila, Ketil Størdal, German Tapia, Heikki Hyöty, Riitta Veijola, Jorma Toppari, Mikael Knip,

Lars C. Stene and Suvi M. Virtanen

**Figure S1.** Selection of MoBa study population.

**Figure S2.** Selection of DIPP study population.

**Statistical analyses - Confounders**

MoBa

Adjusted analyses included the following confounders: maternal pre-pregnancy BMI, mother’s age at delivery, education level (less than high school / high school or college), smoking (no smoking during the pregnancy, stopped during the first trimester, continued after the first trimester), maternal asthma (yes/no), cesarean section (yes/no), parity (number of previous deliveries), child’s preterm birth (before 37 completed weeks), eczema at 6 months (yes/no), and sex of the child (boy/girl).

DIPP

Adjusted analyses included the following confounders: maternal pre-pregnancy body mass index (BMI), mother’s age at delivery, education (no vocational education or university degree, vocational school or course, secondary vocational education, university studies or degree), smoking (no smoking during pregnancy or smoking during pregnancy), maternal asthma (yes/no), cesarean section (yes/no), parity (defined as the number of deliveries), child’s premature birth (yes/no), eczema during the first 6 months (yes/no), and sex of the child (boy/girl).

**Statistical analyses - Absolute risk estimation**

MoBa

Because the MoBa dataset comprises complete-case, non-imputed data, absolute risk profiles were derived via Stata’s post-estimation predictive margins framework. The adjusted baseline absolute risk for each daycare stratum was calculated by holding all individual background confounding covariates at their real-world, sample-observed values while fixing breastfeeding duration at the cohort mean of 9.9 months. To evaluate the absolute trajectory over a continuous 3-month breastfeeding increment, these baseline predictive margins were combined with average marginal effects. This approach calculates the exact, model-adjusted absolute risk changes natively across the daycare strata, utilizing the Delta method to compute 95% confidence intervals (CIs) that account for the model's interaction covariance.

**Table S1.** Interaction between daycare attendance by 18 months of age and total duration of breastfeeding on the risk of developing asthma and its clinical subtypes: unadjusted models in the MoBa cohort. Relative risk (RR) values represent the effect sizes modeled per single 1-month increment of breastfeeding duration. Bf=breastfeeding

|  | No disease  Bf duration months  mean (SD)^α^ | Disease  Bf duration months  mean (SD) ^α^ | RR (95% CI) for breastfeeding | Breastfeeding, daycare interaction P-value |
| --- | --- | --- | --- | --- |
| **Asthma** | | | | |
| Daycare | 9.9 (4.4), N=49,458 | 9.5 (4.6), N=1955 | 0.98 (0.97-0.99) | 0.10 |
| No daycare | 10.0 (4.8), N=20,142 | 9.8 (5.0), N=1003 | 0.99 (0.98-1.00) |  |
| **Nonatopic asthma** | | | | |
| Daycare | 10.1 (4.3), N=32,806 | 9.3 (4.7), N=791 | 0.96 (0.95-0.98) | 0.11 |
| No daycare | 10.2 (4.7), N=12,271 | 9.8 (5.2), N=373 | 0.98 (0.96-1.01) |  |
| **Atopic asthma** | | | | |
| Daycare | 10.1 (4.3), N=32,806 | 9.7 (4.7), N=527 | 0.98 (0.96-1.00) | 0.03 |
| No daycare | 10.2 (4.7), N=12,271 | 10.6 (4.8), N=264 | 1.02 (0.99-1.04) |  |

**Table S2.** Estimated adjusted absolute risk trajectory of the asthma variables associated with a 3-month increase in breastfeeding duration in relation to the mean breastfeeding duration of 9.9 months stratified by daycare category in MoBa dataset.

| **Asthma endpoint & daycare stratum** | **Adjusted RR (95% CI)** | **Adjusted baseline absolute risk (95% CI)** | **Estimated absolute risk following a 3-Month Step-Increase (95% CI)** |
| --- | --- | --- | --- |
| **Asthma** |  |  |  |
| No daycare by 18 months | 1.02 (0.98 – 1.06) | 4.61% (4.32% – 4.90%) | 4.71% (4.24% – 5.18%) |
| Daycare by 18 months | 0.97 (0.92 – 1.02) | 3.85% (3.67% – 4.02%) | 3.75% (3.45% – 4.03%) |
| **Nonatopic Asthma** |  |  |  |
| No daycare by 18 months | 1.01 (0.95 – 1.08) | 2.81% (2.52% – 3.11%) | 2.83% (2.36% – 3.32%) |
| Daycare by 18 months | 0.93 (0.85 – 1.01) | 2.36% (2.20% – 2.25%) | 2.18% (1.91% – 2.47%) |
| **Atopic Asthma** |  |  |  |
| No daycare by 18 months | 1.09 (1.01 – 1.18) | 2.08% (1.82% – 2.33%) | 2.27% (1.84% – 2.69%) |
| Daycare by 18 months | 0.98 (0.91 – 1.05) | 1.61% (1.47% – 1.74%) | 1.57% (1.34% – 1.80%) |

**Table S3.** Interaction between daycare attendance by 18 months in MoBa and 24 months in DIPP and duration of exclusive breastfeeding in the risk of developing asthma. Relative risk (RR) and hazard ratio (HR) values represent the effect sizes modeled per single 1-month increment of breastfeeding duration. Bf=breastfeeding

|  | **MoBa** | | | | **DIPP** | | | |
| --- | --- | --- | --- | --- | --- | --- | --- | --- |
|  | No disease  bf duration months mean (SD) | Disease  bf duration months mean (SD) | Adj. RR (95% CI)* | Adj. p-value for interaction* | No disease  bf duration months mean (SD) | Disease bf duration months mean (SD) | Adj. HR (95% CI) * | Adj. p-value for interaction* |
| **Asthma** | | | | | | | | |
| Daycare | 3.5 (2.0), N=47,590 | 3.3 (2.1), N=1,877 | 0.97 (0.95-1.00) | 0.54 | 1.8 (1.7), N=1,404 | 1.8 (1.8), N=62 | 1.02 (0.87-1.19) | 0.80 |
| No daycare | 3.6 (2.1), N=19,189 | 3.3 (2.1), N=961 | 1.00 (0.97-1.03) |  | 2.1 (1.8), N=1,508 | 2.0 (1.7), N=69 | 0.99 (0.86-1.14) |  |
| **Nonatopic asthma** | | | | | | | | |
| Daycare | 3.5 (2.0), N=31,684 | 3.2 (2.1), N=758 | 0.96 (0.92-0.99) | 0.65 | 1.8 (1.7), N=1,404 | 1.6 (1.8), N=18 | 0.92 (0.68-1.24) | 0.87 |
| No daycare | 3.6 (2.1), N=11,760 | 3.3 (2.1), N=351 | 0.99 (0.94-1.04) |  | 2.1 (1.8), N=1,508 | 1.8 (1.7), N=28 | 0.95 (0.76-1.19) |  |
| **Atopic asthma** | | | | | | | | |
| Daycare | 3.5 (2.0), N=31,684 | 3.2 (2.1), N=512 | 0.96 (0.92-1.00) | 0.09 | 1.8 (1.7), N=1,404 | 2.0 (1.8), N=44 | 1.07 (0.89-1.29) | 0.98 |
| No daycare | 3.6 (2.1), N=11,760 | 3.5 (2.1), N=257 | 1.02 (0.96-1.08) |  | 2.1 (1.8), N=1,508 | 2.3 (1.7), N=37 | 1.07 (0.89-1.28) |  |

*Adjusted for duration of exclusive breastfeeding, daycare attendance, and their interaction, as well as maternal pre-pregnancy BMI, age, education, smoking, asthma, c-section, parity, child’s premature birth, eczema by the first 6 months, and sex.

**Table S4.** Interaction between daycare attendance by 36 months of age (yes/no) and the total duration of breastfeeding in the risk of developing asthma in MoBa and DIPP study cohorts. Relative risk (RR) and hazard ratio (HR) values represent the effect sizes modeled per single 1-month increment of breastfeeding duration. Bf=breastfeeding

|  | **MoBa** | | | | | **DIPP** | | | |
| --- | --- | --- | --- | --- | --- | --- | --- | --- | --- |
|  | No disease bf duration months mean (SD) | Disease bf duration months mean (SD) | Adj. RR (95% CI)* | Adj. p-value for interaction* | | No disease  bf duration months mean (SD) | Disease bf duration months mean (SD) | Adj. HR (95% CI)* | Adj. p-value for interaction* |
| **Asthma** | | | | |  | | | | |
| Daycare | 10.0 (4.4), N=37,481 | 9.6 (4.6), N=1,632 | 1.00 (0.99-1.01) | 0.74 | | 7.6 (5.0), N=1,894 | 6.6 (4.4), N=57 | 0.97 (0.91-1.03) | 0.85 |
| No daycare | 9.8 (4.7), N=2,290 | 9.2 (5.6), N=112 | 1.00 (0.96-1.04) |  | | 8.6 (5.4), N=990 | 7.5 (5.8), N=28 | 0.98 (0.91-1.05) |  |
| **Nonatopic asthma** | | | | | | | | | |
| Daycare | 10.1 (4.4), N=27,712 | 9.6 (4.7), N=740 | 0.99 (0.98-1.01) | 1.00 | | 7.6 (5.0), N=1,894 | 5.6 (4.1), N=17 | 0.93 (0.83-1.05) | 0.69 |
| No daycare | 9.8 (4.7), N=1,558 | 9.2 (5.5), N=51 | 1.00 (0.94-1.05) |  | | 8.6 (5.4), N=990 | 6.9 (5.2), N=12 | 0.96 (0.86-1.08) |  |
| **Atopic asthma** | | | | | | | | | |
| Daycare | 10.1 (4.4), N=27,712 | 9.9 (4.5), N=481 | 1.00 (0.98-1.02) | 0.54 | | 7.6 (5.0), N=1,894 | 7.1 (4.5), N=39 | 0.99 (0.93-1.06) | 0.99 |
| No daycare | 10.0 (4.7), N=1,558 | 9.2 (5.9), N=33 | 1.00 (0.93-1.08) |  | | 8.6 (5.4), N=990 | 8.0 (6.4), N=16 | 0.99 (0.90-1.09) |  |

*Adjusted for total duration of breastfeeding, daycare attendance, and their interaction, as well as maternal pre-pregnancy BMI, age, education, smoking, asthma, c-section, parity, child’s premature birth, eczema by the first 6 months, and sex.

**Table S5.** Interaction between daycare attendance by 18 months of age (yes/no) and the total duration of breastfeeding in the risk of developing asthma in MoBa study cohort. Adjusted relative risk (aRR) values represent the effect sizes modeled per single 1-month increment of breastfeeding duration.

|  | **No disease,**  breastfeeding mean duration (SD) | **Disease,**  breastfeeding mean duration (SD) | **aRR (95% CI)*** | **Adj. p-value for interaction*** |
| --- | --- | --- | --- | --- |
| **Asthma** |  |  |  |  |
| Daycare | 9.9 (4.4), N=44,570 | 9.5 (4.7), N=1,746 | 0.99 (0.98-1.00) | 0.03 |
| no daycare | 10.0 (4.8), N= 17,376 | 9.9 (5.0), N= 861 | 1.01 (1.00-1.03) |  |
| **Nonatopic asthma** |  |  |  |  |
| Daycare | 10.1 (4.3), N=29,764 | 9.4 (4.7) 708 | 0.97 (0.96-0.99) | 0.03 |
| No daycare | 10.3 (4.7), N= 10,737 | 10.1 (5.1) 325 | 1.01 (0.99-1.03) |  |
|  |  |  |  |  |
| **Atopic asthma** |  |  |  |  |
| daycare | 10.1 (4.3), N= 29,764 | 9.7 (4.6), N=474 | 0.99 (0.97-1.01) | 0.02 |
| no daycare | 10.3 (4.7), N= 10,737 | 10.6 (4.8), N= 220 | 1.03 (1.00-1.06) |  |
|  |  |  |  |  |

*Adjusted for total duration of breastfeeding, daycare attendance, and their interaction, as well as household income, maternal pre-pregnancy BMI, age, education, smoking, asthma, c-section, parity, child’s premature birth, eczema by the first 6 months, and sex.

**Table S6.** Daycare attendance by 18 months of age and duration of any breastfeeding and the risk of any asthma and non-atopic and atopic asthma in MoBa research cohort. Relative risk (RR) values represent the effect sizes modeled per single 1-month increment of breastfeeding duration.

|  | | No disease  N (%) or mean (SD)^α^ | Disease  N (%) or mean (SD)^α^ | No disease  N (%) or mean (SD)^γ^ | Disease  N (%) or mean (SD)^γ^ | Unadjusted  RR (95% CI)^α^ | Adjusted RR (95% CI)^γ^* | Unadj. p-value^α^ | Adj. p-value ^γ^* |
| --- | --- | --- | --- | --- | --- | --- | --- | --- | --- |
| **Asthma** | | | | | | | | | |
| Daycare | Yes | 49,458 (96.2) | 1,955 (3.8) | 47,590 (96.2) | 1,877 (3.8) | 0.80 (0.74-0.86) | 0.83 (0.77-0.90) | <0.001 | <0.001 |
|  | No | 20,142 (95.3) | 1,003 (4.7) | 19,189 (95.2) | 961 (4.8) | 1 | 1 |  |  |
| Breastfeeding duration, mo | | 9.9 (4.5) | 9.6 (4.8) | 9.9 (4.5) | 9.6 (4.8) | 0.98 (0.97-0.99) | 1.00 (0.99-1.01) | <0.001 | 0.51 |
| **Nonatopic asthma** | | | | | | | | | |
| Daycare | Yes | 32,806 (97.7) | 791 (2.4) | 31,684 (97.7) | 758 (2.3) | 0.80 (0.71-0.90) | 0.84 (0.74-0.95) | <0.001 | 0.008 |
|  | No | 12,271 (97.1) | 373 (3.0) | 11,760 (97.1) | 351 (2.9) | 1 | 1 |  |  |
| Breastfeeding duration, mo | | 10.1 (4.4) | 9.5 (4.9) | 10.1 (4.4) | 9.5 (4.8) | 0.97 (0.96-0.98) | 0.98 (0.97-1.00) | <0.001 | 0.02 |
| **Atopic asthma** | | | | | | | | | |
| Daycare | Yes | 32,806 (98.4) | 527 (1.6) | 31,684 (98) | 512 (1.6) | 0.75 (0.65-0.87) | 0.77 (0.66-0.89) | <0.001 | 0.001 |
|  | No | 12,271 (97.9) | 264 (2.1) | 11,760 (98) | 257 (2.1) | 1 | 1 |  |  |
| Breastfeeding duration, mo | | 10.1 (4.4) | 9.9 (4.7) | 10.1 (4.4) | 10.0 (4.7) | 0.99 (0.98-1.01) | 1.01 (0.99-1.02) | 0.43 | 0.43 |

**^α^**Based on unadjusted full data set, ^γ^Based on fully adjusted data set including those individuals who had data on all covariates available , *Models for breastfeeding and daycare attendance were mutually adjusted for each other. All models were further adjusted for maternal pre-pregnancy BMI, age, education, smoking, c-section, asthma, parity, child’s premature birth, eczema by the first 6 months, and sex.

|  | No disease  Bf duration  months mean (SD) | Disease  Bf duration  months mean (SD) | HR (95% CI)  for breastfeeding | P-value for interaction |
| --- | --- | --- | --- | --- |
| **Asthma** | | | | |
| Daycare | 7.4 (4.8), N=1,393 | 6.5 (3.8), N=62 | 0.96 (0.91-1.02) | 0.90 |
| No daycare | 8.4 (5.5), N=1,491 | 7.4 (5.4), N=69 | 0.97 (0.92-1.01) |  |
| **Nonatopic asthma** | | | | |
| Daycare | 7.4 (4.8), N=1,393 | 5.6 (3.9), N=18 | 0.91 (0.81-1.02) | 0.81 |
| No daycare | 8.4 (5.5), N=1,491 | 6.4 (4.2), N=28 | 0.92 (0.85-1.00) |  |
| **Atopic asthma** | | | | |
| Daycare | 7.4 (4.8), N=1,393 | 7.0 (3.7), N=44 | 0.98 (0.92-1.05) | 0.69 |
| No daycare | 8.4 (5.5), N=1,491 | 8.3 (6.4), N=37 | 1.00 (0.94-1.06) |  |

**Table** **S7.** Interaction between daycare attendance by the age of 24 months and the total duration of breastfeeding in the risk of developing asthma in DIPP research cohort based on unadjusted analyses. Hazard ratio (HR) values represent the effect sizes modeled per single 1-month increment of breastfeeding duration. Bf=breastfeeding

**Table S8.** Interaction between the three-level daycare attendance variable (daycare by the age of 12 months, daycare by the age of 13 to 24 months, no daycare by the age of 24 months) and total duration of breastfeeding on the risk of developing asthma in DIPP study cohort. Based on unadjusted analyses. Hazard ratio (HR) values represent the effect sizes modeled per single 1-month increment of breastfeeding duration. Bf=breastfeeding

|  | No disease  Bf duration  months mean (SD) | Disease  Bf duration  months mean (SD) | HR (95% CI) for breastfeeding | P-value for interaction |
| --- | --- | --- | --- | --- |
| **Asthma** | | | | |
| Daycare by 12 months | 6.8 (4.5), N=426 | 4.4 (4.0), N=17 | 0.86 (0.75-0.99) | 0.22 |
| Daycare by 13-24 months | 7.7 (4.9), N=967 | 7.4 (3.4), N=45 | 0.99 (0.93-1.05) |  |
| No daycare by 24 months | 8.4 (5.5), N=1,491 | 7.4 (5.4), N=69 | 0.97 (0.92-1.01) |  |
| **Nonatopic asthma** | | | | |
| Daycare by 12 months | 6.8 (4.5), N=426 | 6.1 (5.1), N=7 | 0.97 (0.81-1.15) | 0.71 |
| Daycare by 13-24 months | 7.7 (4.9), N=967 | 5.2 (3.0), N=11 | 0.87 (0.75-1.03) |  |
| No daycare by 24 months | 8.4 (5.5), N=1,491 | 6.4 (4.2), N=28 | 0.92 (0.85-1.00) |  |
| **Atopic asthma** | | | | |
| Daycare by 12 months | 6.8 (4.5), N=426 | 3.2 (2.6), N=10 | 0.76 (0.60-0.95) | 0.06 |
| Daycare by 13 to 24 months | 7.7 (4.9), N=967 | 8.1 (3.3), N=34 | 1.01 (0.95-1.08) |  |
| No daycare by 24 months | 8.4 (5.5), N=1,491 | 8.3 (6.2), N=37 | 1.00 (0.94-1.06) |  |

|  | | No asthma  N (%) or mean (SD) | Asthma  N (%) or mean (SD) | Unadjusted HR (95% CI) | Adjusted HR (95% CI)* | Unadj. p-value | Adj. p-value* |
| --- | --- | --- | --- | --- | --- | --- | --- |
| **Asthma** | | | | | | | |
| Daycare | Yes | 1,393 (95.7%) | 62 (4.3%) | 0.96 (0.68-1.37) | 0.87 (0.61-1.25) | 0.83 | 0.45 |
|  | No | 1,491 (95.6%) | 69 (4.4%) | 1 | 1 |  |  |
| Breastfeeding duration, mo. | | 7.9 (5.2 SD) | 7.0 (4.7 SD) | 0.96 (0.93-1.00) | 0.97 (0.93-1.01) | 0.05 | 0.12 |
| **Nonatopic asthma** | | | | | | | |
| Daycare | Yes | 1,393 (98.7%) | 18 (1.3%) | 0.69 (0.38-1.25) | 0.64 (0.35-1.17) | 0.22 | 0.15 |
|  | No | 1,491 (98.2%) | 28 (1.8%) | 1 | 1 |  |  |
| Breastfeeding duration, mo. | | 7.9 (5.2 SD) | 6.1 (4.1 SD) | 0.92 (0.86-0.99) | 0.94 (0.87-1.00) | 0.02 | 0.06 |
| **Atopic asthma** | | | | | | | |
| Daycare | Yes | 1,393 (96.9%) | 44 (3.1%) | 1.27 (0.82-1.96) | 1.19 (0.75-1.88) | 0.29 | 0.45 |
|  | No | 1,491 (97.6%) | 37 (2.4%) | 1 | 1 |  |  |
| Breastfeeding duration, mo. | | 7.9 (5.2 SD) | 7.6 (5.0 SD) | 0.99 (0.95-1.03) | 1.00 (0.95-1.04) | 0.56 | 0.86 |

**Table S9.** Daycare attendance by the age of 24 months, duration of any breastfeeding and risk of asthma in DIPP research cohort. Hazard ratio (HR) values represent the effect sizes modeled per single 1-month increment of breastfeeding duration.

*Models for breastfeeding and daycare attendance were mutually adjusted for each other. All models were further adjusted for maternal pre-pregnancy BMI, age, education, smoking, c-section, asthma, parity, child’s premature birth, eczema by the first 6 months, and sex.
